# Evaluation of the Impact of the LPBF Manufacturing Conditions on Microstructure and Corrosion Behaviour in 3.5 wt.% NaCl of the WE43 Magnesium Alloy

**DOI:** 10.3390/ma18153613

**Published:** 2025-07-31

**Authors:** Jorge de la Pezuela, Sara Sánchez-Gil, Juan Pablo Fernández-Hernán, Alena Michalcova, Pilar Rodrigo, Maria Dolores López, Belén Torres, Joaquín Rams

**Affiliations:** 1 Department of Applied Mathematics, Materials Science and Engineering and Electronics Technology, ESCET, Universidad Rey Juan Carlos, 28933 Mostoles, Spain; jorge.delapezuela@urjc.es (J.d.l.P.); sara.sgil@urjc.es (S.S.-G.); juanpablo.fernandez@urjc.es (J.P.F.-H.); pilar.rodrigo@urjc.es (P.R.); mariadolores.lopez@urjc.es (M.D.L.); belen.torres@urjc.es (B.T.); 2 Department of Metals and Corrosion Engineering, University of Chemistry and Technology, Technická 5, Prague 6, 166 28 Prague, Czech Republic; alena.michalcova@vscht.cz; 3 Instituto de Investigación de Tecnologías para la Sostenibilidad, Universidad Rey Juan Carlos, C/Tulipán s/n, 28933 Móstoles, Spain

**Keywords:** magnesium, WE43, additive manufacturing, laser powder bed fusion, corrosion

## Abstract

This work expands the processing window of the laser powder bed fusion (LPBF) processing of WE43 magnesium alloy by evaluating laser powers and scanning speeds up to 400 W and 1200 mm/s, and their effect on densification, microstructure, and electrochemical performance. Relative density of 99.9% was achieved for 300 W and 800 mm/s, showing that the use of high laser power is not a limitation for the manufacturing of Mg alloys, as has been usually considered. Microstructural characterisation revealed refined grains and the presence of RE-rich intermetallic particles, while microhardness increased with height due to thermal gradients. Electrochemical testing in 3.5 wt.% NaCl solution, a more aggressive media than those already used, indicated that the corrosion of samples with density values below 99% is conditioned by the porosity; however, above this value, in the WE43, the corrosion evolution is more related to the microstructure of the samples, according to electrochemical evaluation. This study demonstrates the viability of high-energy LPBF processing for WE43, offering optimised mechanical and corrosion properties for biomedical and structural applications.

## 1. Introduction

Magnesium is the lightest structural material due to its density of 1.8 g/cm^3^. Mg alloys have a high specific strength, high specific stiffness, high conductivity, are biocompatible, and recyclable. Magnesium and magnesium alloys are of interest in many sectors, such as transport or aerospace [1], where they can substitute aluminium and titanium alloys or steels, reducing the weight of the structures [1,2,3,4]. They are also relevant materials as orthopedic implants because their mechanical properties are close to those of the human bones, and their biodegradation in the body [5].

Laser powder bed fusion (LPBF) is an additive manufacturing (AM) technique that relies on the layer-by-layer deposition of metallic powder, which is selectively melted using a laser according to a predefined pattern. This technique allows for a high degree of customisation in the production of parts and can be used to create complex geometries, such as lattice or cellular structures, or that mimic natural shapes [6]. Additionally, it enables the production of net-shape parts, i.e., parts that come out of the process with their final geometry, requiring only minimal post-processing to perfect the surface. This capability not only expands design possibilities but also reduces manufacturing costs and waste of material. LPBF locally melts the powder, and the fast cooling and solidification of the material causes a complex microstructure [6,7]. As a result, small grains are formed [7], and the material shows an anisotropic microstructure [8], which causes a complex mechanical behaviour [9].

Mg has a high chemical activity due to its low electrochemical potential, and because the surface oxide layer has poor protective capacity, limiting its applications [10,11,12]. To reduce it, magnesium is commonly alloyed with other elements such as Al, Si, Zn, or Ca. Also, rare earths (RE) such as Y, Nd, Gd, or Zr are used because they modify the electrochemical behaviour of the alloy. Zhang et al. [13] studied the effect of the Y concentration and concluded that increasing the Y content in the alloy decreases its reactivity because the Mg_24_Y_5_ phases formed exhibit cathodic behaviour against the α-Mg one. However, they found that pitting may occur at the interface of both phases due to the formation of micro-galvanic couples. Coy et al. [14] studied that Zr-rich areas had a much higher potential (+170 mV) than the Mg matrix and caused localised corrosion. Song et al. [15] concluded that Zr-rich areas located within the grains have higher corrosion resistance than Zr-rich grain boundaries.

Y, Gd, and Zr also improve the mechanical properties and creep resistance, both at room temperature and at elevated temperatures, because they promote solid solution strengthening and precipitation hardening mechanisms [16]. Gao et al. [17] studied the influence of Gd content on the material’s strength, increasing the strength while increasing the concentration of Gd. In the case of Yttrium (Y), Su et al. conducted a study that determined that the use of Y as an alloying element increased hardness as well as creep resistance through the precipitation of intermetallic phases that hinder dislocation movement [18]. Regarding Zr, it is the most efficient grain refiner, which means it enhances the mechanical properties of the material [19].

The LPBF of Mg alloys presents several challenges, mainly because of the high vapor pressure of Mg and of some of the most common alloyants. In Mg alloys, an excess of energy produces alloy elements vaporisation [20], causing porosity and keyholes [21]. Moreover, a lack of dimensional accuracy may be present due to the fusion of the surrounding powder caused by heat transfer [22,23]. On the contrary, if insufficient energy is applied, the powder may not melt [24], resulting again in porosity. Also, lack of fusion, thermal defects, sputtering, and smog appear during the process [25,26].

Several authors have investigated the processing parameters of the WE43 alloy within a laser power range of 60–240 W, aiming to achieve high levels of densification [27,28]. Relative densities of up to 99.9% have been reported when employing low scanning speeds and thin powder layers [29,30]. However, the effects of higher laser powers combined with increased layer thickness remain largely unexplored, representing a gap in the current literature. The corrosion behaviour of LPBF-processed WE43 alloy in low-chloride environments—such as Hank’s solution and 0.1 M NaCl—has been investigated primarily in samples with relative densities below 99.7%. However, there is a lack of studies involving denser specimens processed under a broader range of manufacturing parameters and exposed to highly chlorinated media. Furthermore, the corrosion mechanisms and their evolution over time remain insufficiently explored. Yin et al. [31] found that increased layer thickness reduces corrosion resistance in Hank’s solution. Grain growth and elemental segregation further degrade performance [32]. Benn et al. [32] reported severe pitting after chemical polishing, while machining improved corrosion behaviour, consistent with results in 0.1 M NaCl [33]. However, these studies achieved densities below 99.7% and used less aggressive environments than standard seawater (0.6 N NaCl).

On the other hand, dense samples have been obtained by postprocessing with HIP [27] or through the optimisation of the manufacturing parameters [34], but these authors have not studied the corrosion behaviour of the alloys.

This study studies the Laser Powder Bed Fusion (LPBF) fabrication of WE43 alloy, with a particular emphasis on the impact of high scanning speeds (800–1200 mm/s) in combination with a broad power range (150–400 W). To enhance process efficiency, higher scanning speeds reduce manufacturing time, thereby increasing cost-effectiveness. However, a knowledge gap remains regarding the influence of microstructural variations and fabrication-induced defects on the material’s properties. Additionally, there is a lack of studies on the electrochemical behaviour of this alloy in highly aggressive environments, such as marine conditions (3.5 wt.% NaCl). A comprehensive microstructural and corrosion analysis under various processing conditions would provide deeper insights into the mechanical and electrochemical performance of WE43, facilitating its optimisation for demanding applications.

## 2. Materials and Methods

### 2.1. Materials and Process Parameters

**Figure 1:**
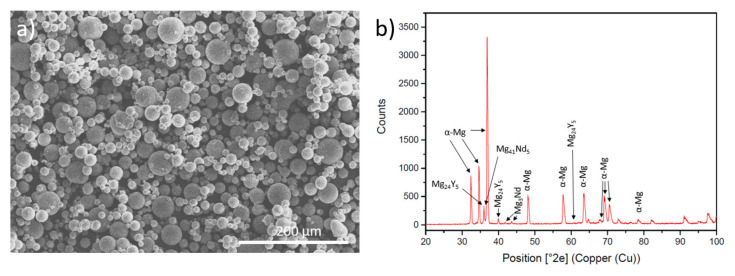
(**a**) Initial SEM morphology WE43 powder; (**b**) XRD of WE43 magnesium alloy powder.

Table 1 shows the nominal composition of the WE43 powder (NMD New Materials Development GmbH, Rosenheim, Germany). The particles were spherical with a diameter between 15 and 53 microns (Figure 1a).

The initial composition of the powder was characterised by X-ray diffractometry (XRD) to identify the phases present in the powder (Figure 1b). It can be observed that, apart from the α-Mg, Mg is found forming intermetallic compounds with neodymium and yttrium.

The Renishaw AM400 LPBF equipment (Wotton-under-Edge, UK) was used. It has a fiber laser with a power of 400 W with a laser spot of 70 microns. The reduced volume build (RBV) system was used. During manufacturing, oxygen was kept below 200 ppm.

**Figure 2:**
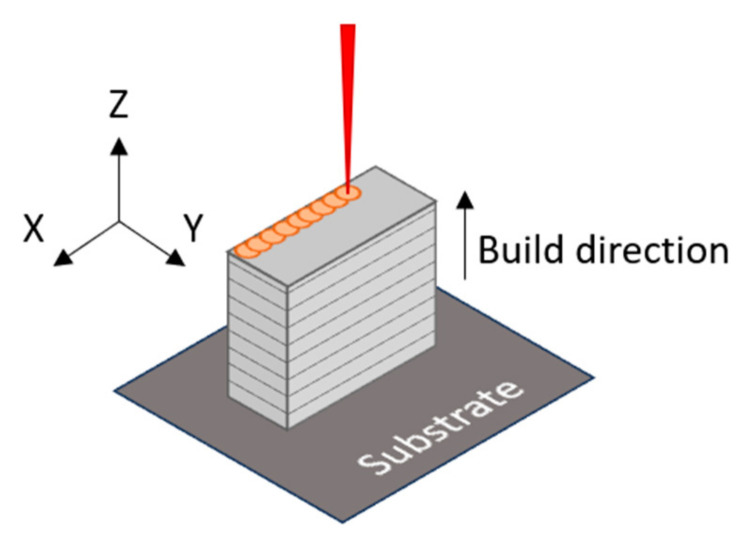
Scheme of build direction.

The specimens with dimensions 1.5 × 1.5 × 0.5 cm^3^ were manufactured with a layer height of 30 µm, a 90 µm hatch, a meander pattern and 67° rotating angle between layers. Figure 2 shows a scheme of the LPBF manufacturing process where the previous parameters are indicated, as well as the building direction of the samples. The power and scan speed conditions used are shown in Table 2.

**Figure 3:**
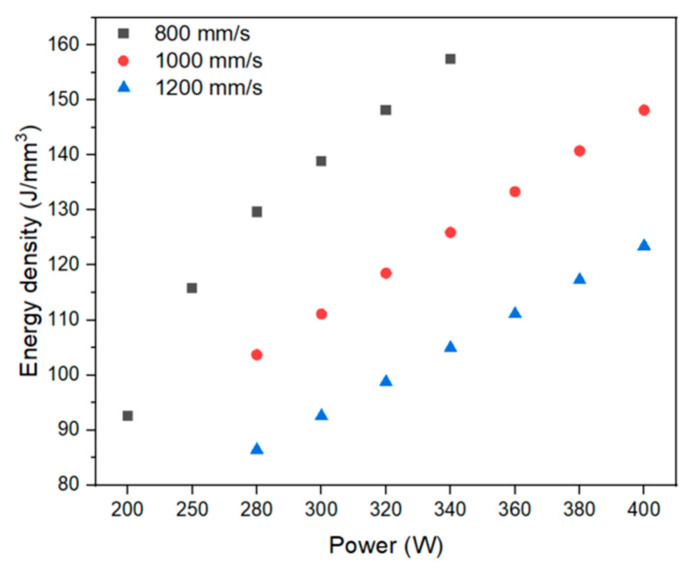
Energy density (J/mm^3^) vs. Power (W) of manufacturing conditions.

The energy parameters, as well as the energy densities for each manufacturing condition, are shown in Figure 3. Equation (1) was used to determine the applied energy density (*E*) using the laser power (*P*), layer thickness (*t*), scanning speed (*v*), and hatch (*h*).

(1) $$E=\frac{P}{v\cdot h\cdot t}.$$

### 2.2. Microstructural Characterisation

Microstructural analysis was performed using both optical and electron microscopy techniques. Optical microscopy (OM) was conducted on a Leica DMR system (Wetzlar, Germany), scanning electron microscopy (SEM) was carried out on a Hitachi S-3400N (Tokyo, Japan), and energy-dispersive X-ray spectroscopy (EDS) with a AXS Xflash Detector 5010 (Bruker, Billerica, MA, USA). Porosity was quantified by image at different depths of the samples ensure representative data across the entire volume. Sample preparation involved sectioning, embedding in resin, mechanical polishing down to 1 µm, and etching with a Nital solution for 40 s. Phase identification was performed by X-ray diffraction (XRD) using a CuKα radiation source (λ = 1.54056 Å). For transmission electron microscopy (TEM), thin foils were prepared by polishing with 0.5 µm SiC papers using a Leica EM-TXP, followed by final thinning with a Leica RES102 ion mill (Leica, Wetzlar, Germany) at 5 kV and a 5° incidence angle. TEM observations were conducted on a JEOL 2200 FS (Tokyo, Japan). Electron backscatter diffraction (EBSD) analysis was performed on an FEI Quanta 3D DualBeam field-emission SEM (Hillsboro, OR, USA) equipped with a TSL/EDAX Hikari EBSD detector (AMETEK, Inc., Berwyn, PA, USA) on ion-polished samples.

Vickers microhardness measurements were carried out using an Innovatest Falcon 500 tester (Maastricht, The Netherlands) applying a 100 g load (HV0.1) for 10 s with ten indentations per condition.

### 2.3. Corrosion Test

Electrochemical characterisation of the WE43 alloy specimens was carried out using an Autolab PGStat302N potentiostat (Metrohm AG, Herisau, Switzerland) in the conventional three-electrode configuration. The samples acted as the working electrode, the reference electrode was silver/silver chloride (Ag/AgCl, 3 M KCl), and the counter electrode was a platinum wire. Tests were conducted in a 3.5 wt.% NaCl aqueous solution at room temperature on ground surfaces. The open circuit potential (OCP) was monitored for 1 h to allow stabilisation of the corrosion potential (E_corr_) before initiating the electrochemical measurements.

Linear polarisation resistance (LPR) tests were performed following the ASTM G59 standard [35] (±10 mV around the OCP at 1 mV/s). Measurements were taken after immersion times of 1, 6, 24, 48, 72, and 96 h, with three replicates per condition to ensure reproducibility. Anodic–cathodic polarisation measurements were performed at 1 mV/s from −400 mV to 600 mV with respect to E_corr_.

Electrochemical impedance spectroscopy (EIS) was conducted by applying a 10 mV sinusoidal voltage at E_corr_, from 10^5^ to 10^−2^ Hz with seven data points per decade. The impedance spectra were fitted to equivalent electrical circuits using FRA software Autolab Nova 2.1 (Utrecht, The Netherlands).

## 3. Results and Discussion

### 3.1. Microstructure and Defects

**Figure 4:**
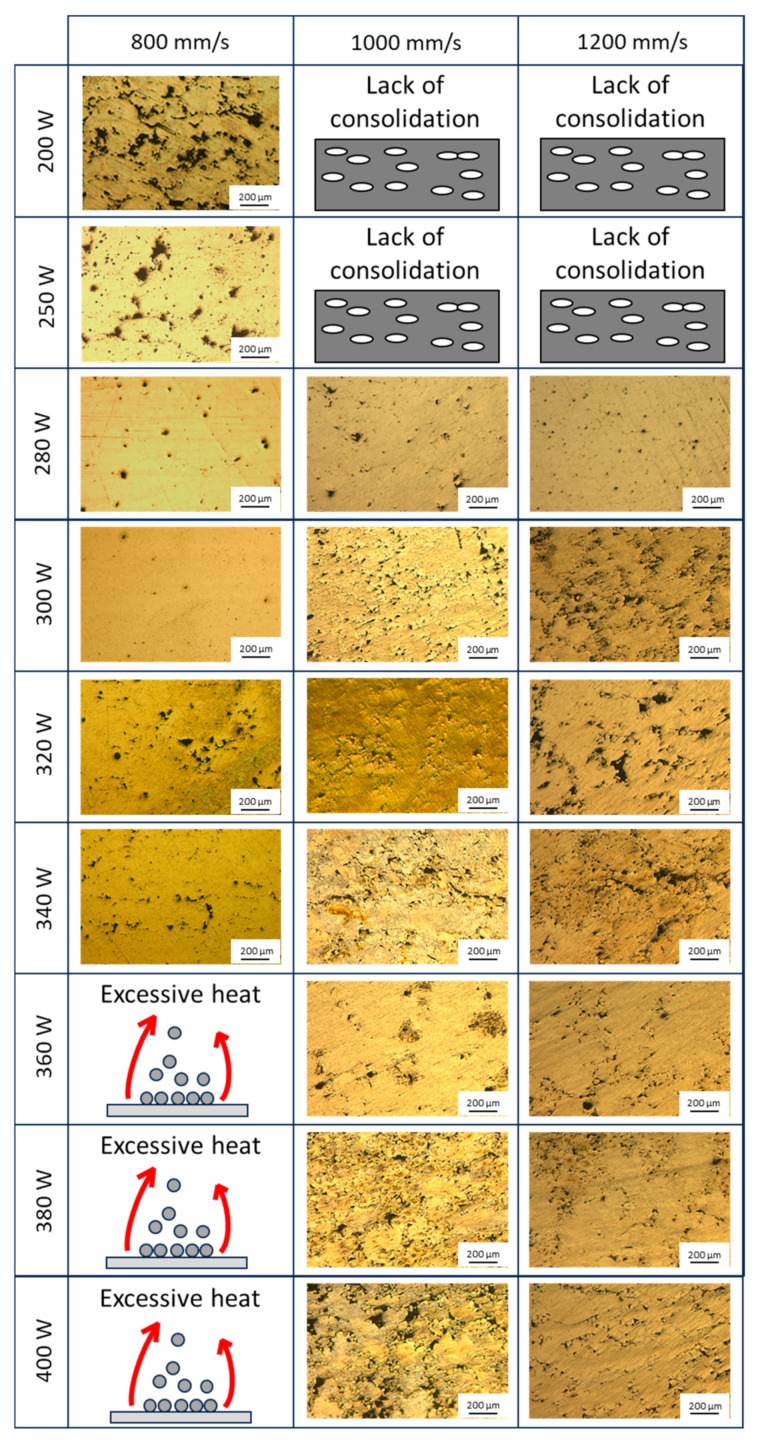
Comparison between the different microstructures obtained under the different conditions.

Figure 4 shows the microstructures of different specimens fabricated with power levels ranging from 200 W to 400 W and scanning speeds between 800 mm/s and 1200 mm/s. Fixing the scanning speed at 800 mm/s, the microstructure obtained at a laser power of 200 W exhibits irregular porosity, which may indicate a lack of material consolidation due to insufficient energy input to achieve proper melting. With an increase in power to 250 W, a reduction in irregular porosity is observed, along with the appearance of small regions of spherical porosity. This 25% increase in power enhances material consolidation compared to the 200 W sample, although full densification is not yet achieved. At 280 W, spherical porosity becomes more evident, likely due to argon gas entrapment during processing. At 300 W, a homogeneous microstructure with high densification is attained. However, increasing the power to 320 W and 340 W results in a heterogeneous distribution of porosity, possibly due to instabilities in the melt pool caused by the elevated energy input. At power levels exceeding 340 W, up to 400 W, the samples exhibit a lack of consolidation due to excessive energy input, leading to substantial material evaporation during processing. This excessive vaporisation results in the formation of a plume of suspended powder, which diminishes laser efficiency and adversely affects the stability of the melting process. This can be explained by the appearance of instabilities in the molten pools caused by excessive energy input and the evaporation of Mg [29,36].

When increasing the scanning speed to 1000 mm/s, the applied energy is reduced due to a shorter thermal interaction between the laser and the material. Consequently, at power levels of 200 W and 250 W, a generalised lack of consolidation is observed, preventing the proper fabrication of the samples. Increasing the power to 280 W results in improved material consolidation; however, porosity and some lack of fusion defects persist. At 300 W, a fully consolidated sample is obtained, but the microstructure exhibits homogeneously distributed spherical porosity. With a further increase to 320 W, material consolidation is maintained, and the amount of spherical porosity decreases throughout the microstructure. At 340 W, lack-of-fusion defects begin to appear, as evidenced by the presence of irregular porosity. At 360 W, an overall reduction in defects is observed compared to the previous power level, leading to higher material densification. However, when increasing the power to 380 W and 400 W, the number of defects increases due to excessive heat accumulation, which may have induced instabilities in the melt pool.

When increasing the laser scanning speed to 1200 mm/s, similar to the previous case, a lack of sample consolidation is observed at power levels of 200 W and 250 W due to insufficient energy input to adequately melt the powder. At 280 W, the sample achieves consolidation with a high degree of densification, as evidenced by the presence of small, homogeneously distributed spherical pores. However, as the power increases to 300 W, irregular defects begin to appear, which persist at power levels of 320 W and 340 W. Notably, defect formation appears to decrease at 360 W, with fewer irregular defects and a greater presence of spherical porosity. At 380 W and 400 W, porosity defects are observed, distributed homogeneously throughout the microstructure.

**Figure 5:**
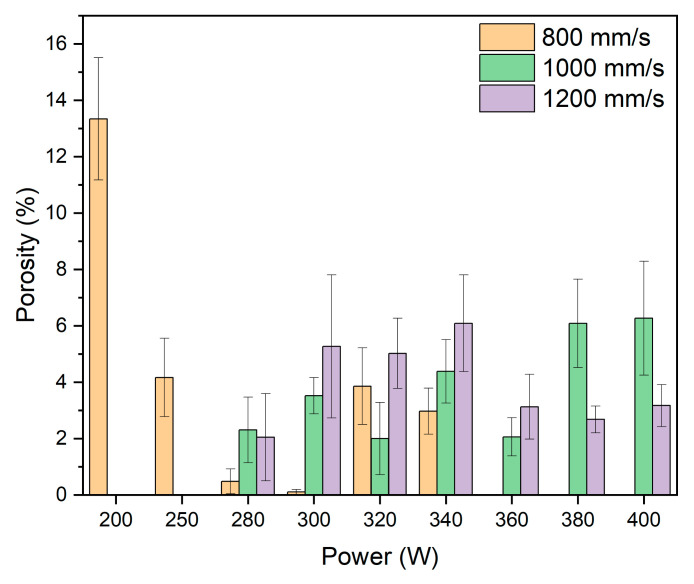
Porosity (%) vs. Power (W) of samples manufactured with different powers and scan speeds.

The porosity values measured through image analysis of the samples are shown in Figure 5. It is observed that at 200 W, the applied energy density is low (<100 J/mm^3^), resulting in a porosity greater than 13%. This value leads to a lack of consolidation due to poor material fusion. At 250 W and 280 W, the energy density progressively increases, thereby achieving higher material densification with a significant reduction in porosity. However, in the case of 280 W, when the scanning speed is increased to 1000 mm/s and 1200 mm/s, the porosity exceeds the values obtained at 800 mm/s. In the power range between 300 W and 340 W, the energy density values range from 100 to 140 J/mm^3^, depending on the scanning speed. At 300 W, significant differences are observed, as the porosity value at 800 mm/s is 0.1%, at 1000 mm/s it is 3.5%, and at 1200 mm/s it is 5.5%. When the power is increased to 320 W, a more pronounced increase is noted at 800 mm/s compared to the previous power, while at the other speeds, a reduction in porosity is observed. At 340 W, the porosity slightly decreases at 800 mm/s, while at the other two speeds, the porosity increases slightly. For powers between 360 W and 400 W, the samples at 800 mm/s are not represented due to a lack of material consolidation during manufacturing. At 360 W, the porosity decreases at 1000 mm/s and 1200 mm/s compared to the previous powers. However, when the power is increased to 380 W and 400 W, the values at 1000 mm/s increase considerably compared to the previous power but remain stable. In the case of 1200 mm/s, there are no significant changes in porosity, with results remaining stable.

These results demonstrate that dense parts can be obtained by applying high power and speed, obtaining values that are comparable or better to studies that use lower power and speed ranges. Suchý et al. [28] used power and speed ranges of 125–225 W and 500–700 mm/s, respectively, achieving a maximum densification of 99.5% for 175 W and 700 mm/s and 50 µm layers. Bär et al. [34] achieved a 99.9% density using 200 W and 700 mm/s, with a layer height of 40 microns, while Zumdick et al. [30] achieved the same density using the same parameters of 200 W and 700 mm/s, but with a layer height of 30 microns. Maintaining the power but increasing speed reduced the density to 99.6% [27]. Therefore, we prove that the density can be increased by increasing the laser power to 300 W without causing the vaporisation of alloying elements, which is the main issue claimed by the different authors that limits the use of high laser power.

**Figure 6:**
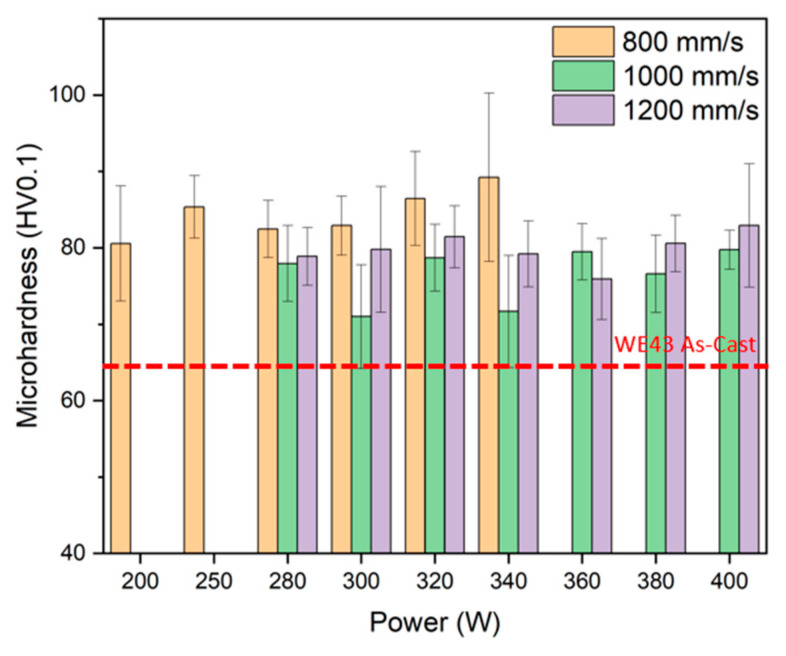
Vickers microhardness HV0.1 of samples manufactured with different powers and scan speeds.

The Vickers microhardness values obtained for the different manufacturing conditions are shown in Figure 6. In all cases, the values are higher than those referred to for the WE43 casting alloy [36]. The higher hardness is caused by the more refined microstructures resulting from the rapid cooling of the layers. For a constant speed of 800 mm/s, the microhardness increases with the increase in power, because of the better consolidated structure, as seen in samples fabricated at lower power levels [37], and because of the faster cooling rate. In the case of the samples at higher speed, the hardness is nearly constant within the uncertainty of the measurement, being generally higher for the fastest scanning speed. Shi et al. [38] reported a microhardness of 84 HV0.2 under processing conditions of 200 W power and a scanning speed of 900 mm/s. Inversely, Suchy et al. [39] documented a microhardness range of 35–45 HV0.01, depending on the applied power. Therefore, we show that higher speeds are required for achieving high hardness values, but that speeds in excess of 800 mm/s did not lead to higher hardness values, suggesting that the cooling speed is not driven by the laser speed but by the thermal conductivity of the alloy used and the density of the material manufactured.

**Figure 7:**
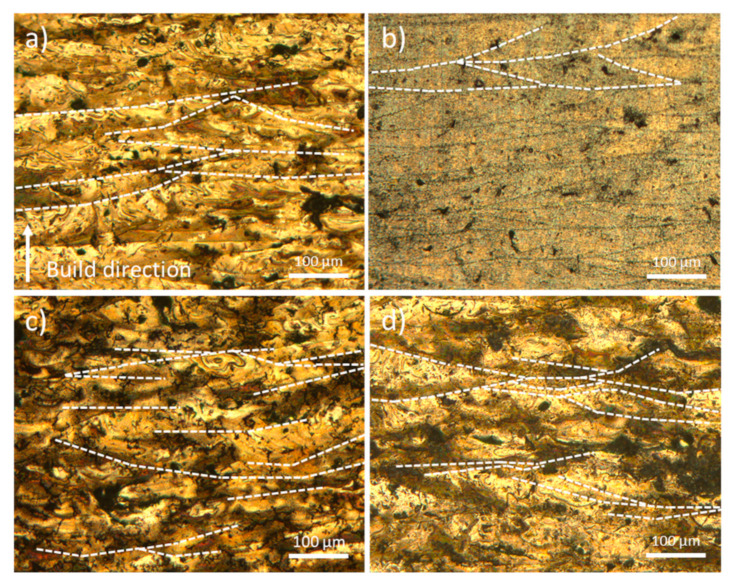
OM of the XZ plane of the samples manufactured at 800 mm/s with (**a**) 280 W; (**b**) 300 W; (**c**) 320 W; (**d**) 340 W.

Figure 7 presents metallographic cross-sections along the vertical XZ plane of samples manufactured at 280 W, 300 W, 320 W, and 340 W, using a constant scanning speed of 800 mm/s. In Figure 7a, the presence of Marangoni convection currents within the molten pools can be observed, indicative of thermocapillary flow driven by surface tension gradients. The sample produced at 300 W (Figure 7b) exhibits the most optimal processing conditions, characterised by a low defect density and a high degree of densification. Accordingly, this condition was selected for subsequent microstructural analysis. With increasing laser power (Figure 7c,d), the residence time of the molten pool increases, promoting the evaporation of volatile elements, such as magnesium. This leads to increased instability within the melt pool, resulting in the formation of internal convective flows that entrap argon gas bubbles and voids caused by elemental evaporation. Consequently, lack of fusion and interlayer porosity defects develop, which adversely affect the cooling dynamics and alter the microstructural evolution of the solidified material [29,40].

**Figure 8:**
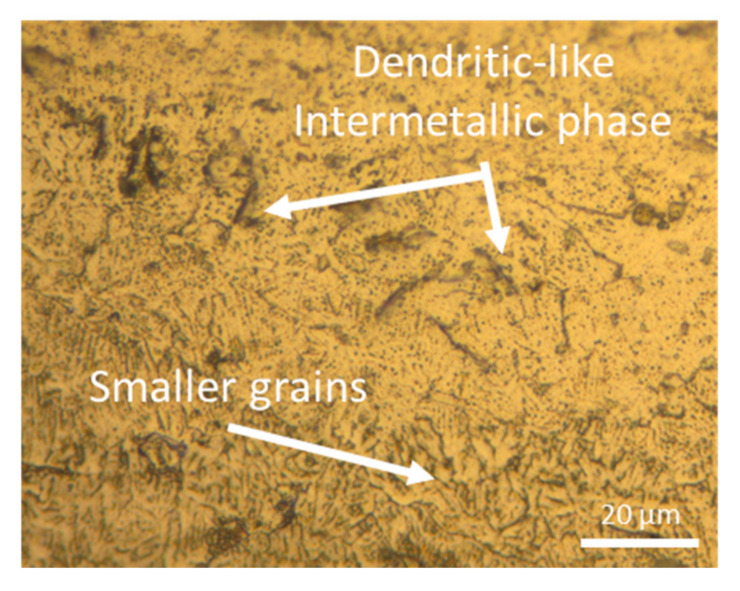
OM magnification of the molten bath limit of as-built sample manufactured with 300 W and 800 mm/s.

Figure 8 shows a magnified view of the microstructure corresponding to the sample produced at 300 W. In this figure, it can be observed the magnification of a melt pool boundary, where small grains appear due to the rapid solidification of the melt pool. Additionally, dendritic shapes corresponding to intermetallic compounds are also visible. The formation of these types of structures is characteristic of eutectic alloys, but it should not occur in the WE43 alloy, as explained by Bär et al. [34] in their study. They indicate that these dendritic or lamellar structures initiate in the partially melted zone, forming an interdendritic liquid enriched with solute elements. In this alloy, dendrites do not form eutectics due to the low content of solute alloying elements and the rapid solidification of the LPBF process. Also, Gianoglio et al. [41] found broken lines formed by precipitates in a laser-melted Al-3Er alloy, similar to the dendritic-like intermetallic phases of Figure 7. They concluded that the columnar crystals of the primary phase grow epitaxially when solidification starts, and eutectic phases appear between them because of lateral rejection of solute. So, the growth direction of these dendrites or lamellae follows the heat dissipation direction in each melt pool.

**Figure 9:**
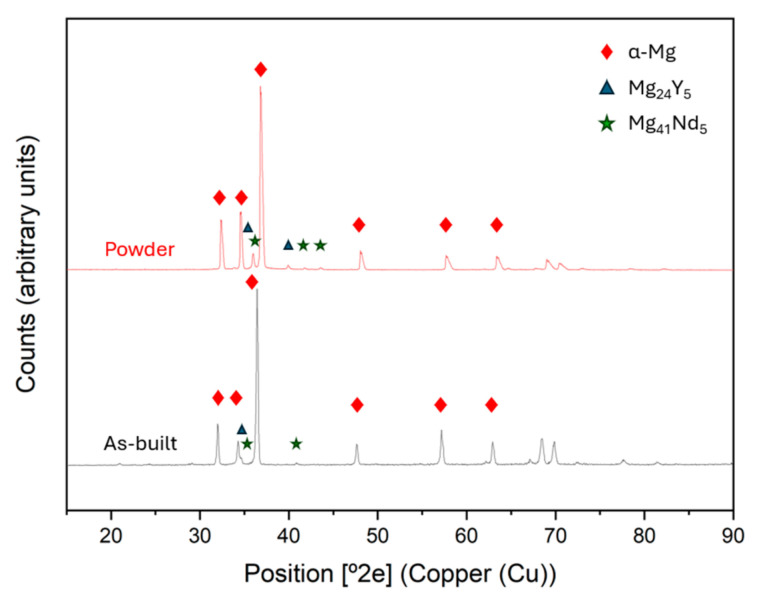
XRD comparison between the initial powder and as-built sample (300 W, 800 mm/s).

Figure 9 shows the XRD of the starting powder, as well as the condition of maximum densification of the material. Although they show almost no differences, the spectrum of the as-built material shows a lower amount of Mg_41_Nd_5_. This may be due to the dissolution of the intermetallic compound because of the fusion of the material during the manufacturing process. Yang et al. [26] studied that the absence of intermetallic phases or precipitates could be caused by the rapid cooling rate of the LPBF process. Also, this promotes the solid solution of alloy elements and reduces or suppresses the formation of secondary phases. On the other hand, it can be seen in Figure 9 that the diffraction peaks corresponding to as-built spectra are positively shifted compared to powder spectra. This phenomenon appears because of the previously mentioned cooling rates of the process, which improve solid solubility of RE elements in the alpha-Mg matrix, so the lattice constant increases, resulting in a decrease in the diffraction angle [42].

**Figure 10:**
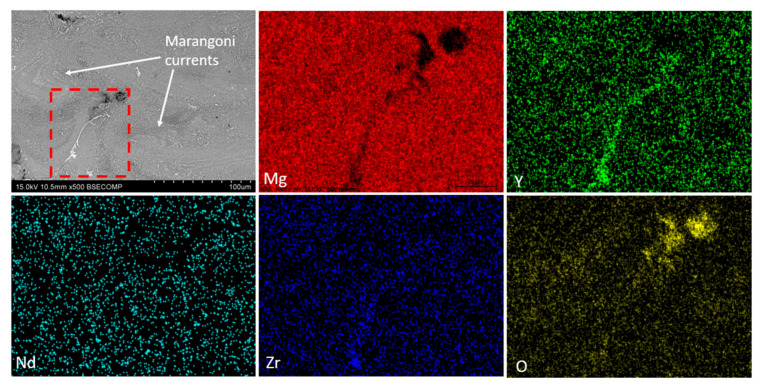
SEM-BSE image and EDX map of the distribution of elements of in the as-built sample (300 W, 800 mm/s).

**Figure 11:**
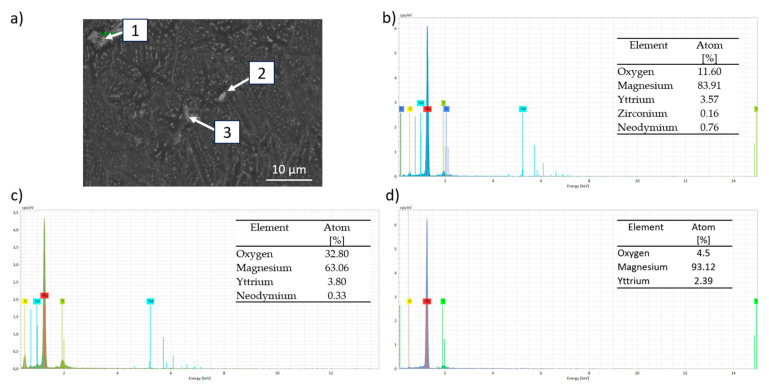
Compositional EDS analyses by EDS of the different intermetallic phases of the as-built sample (300 W, 800 mm/s): (**a**) SEM-BSE image; (**b**) point 1; (**c**) point 2; (**d**) point 3.

Figure 10 shows the SEM-BSE micrograph where Marangoni currents can be observed because of the material remaining molten for a longer time due to the overlap of the laser passes [43]. The compositional analysis shows that oxygen is present throughout the structure of the material, forming MgO. Figure 11 shows an EDS elemental analysis of the microstructure of the dense sample. It can be observed that the map indicates the presence of the α-Mg phase as the matrix, with the remaining alloying elements forming oxides and intermetallic phases. Figure 11 also presents the compositional analysis of the precipitates where three analyses are distinguished in Figure 11a: point 1 (Figure 11b) corresponds to a mixed Y-Nd-Zr oxide; point 2 (Figure 11c) shows a mixed Y-Nd oxide; while point 3 (Figure 11d) reveals the presence of the Mg-Y intermetallic, with the most common composition being Mg_24_Y_5_. The presence of this oxide can be attributed to two main reasons, as described in the literature. Salehi et al. [44] associate this phenomenon with the presence of residual oxygen from the inert gas during manufacturing. Another reason was proposed by Dreizin et al. [45], where it was indicated that the oxygen comes from the passive layer of the powder, most likely from the irregular particle shapes. Additionally, the presence of intermetallic precipitates is related to the higher affinity of rare earth elements (RE) for oxygen compared to magnesium (Mg) [46].

**Figure 12:**
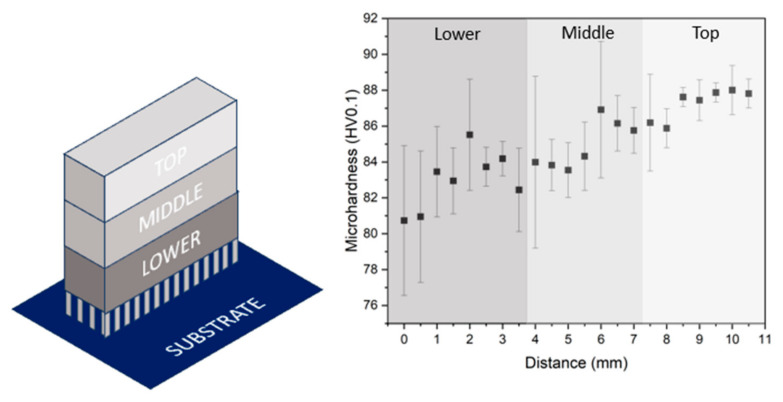
Microhardness evolution from the lower zone to the top one of the as-built sample (300 W, 800 mm/s).

The hardness distribution in the sample manufactured under the optimal condition, i.e., 300 W and 800 mm/s, has been studied. Figure 12 shows the microhardness results along the XZ plane at different distances from the building platform. It can be seen that the hardness in the parts increases with the distance to the substrate. Due to the layer-by-layer build-up of the sample, the lower part has been submitted to more heating and cooling cycles than the top part, and this could have an effect on the microstructure of the sample. To evaluate this, the cross section of the samples on its XY plane was analysed at different heights as shown in Figure 12. For each height, a TEM microscopy analysis has been carried out, and the precipitates have been determined. In addition, an EBSD analysis of the areas adjacent to each of the sections was also carried out.

**Figure 13:**
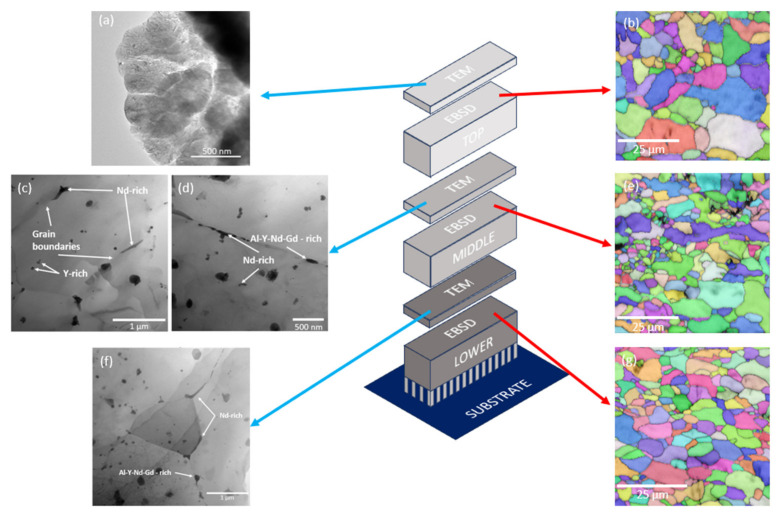
Graphical scheme of the slices, TEM micrographs, and EBSD maps of each section: (**a**) TEM micrograph and (**b**) EBSD map of top section; (**c**,**d**) TEM micrographs and (**e**) EBSD map of middle section; (**f**) TEM micrograph and (**g**) EBSD map of lower section.

Figure 13 illustrates the methodology employed for the microstructural analysis of the sample using TEM and EBSD on cross-sections at different depths (top, middle, and bottom), as previously indicated in Figure 12. All visible precipitates observed in the TEM micrographs (Figure 13c–f) were analysed using energy-dispersive X-ray spectroscopy (EDS). However, for clarity, only the most representative and compositionally distinct precipitates are highlighted in the figure. The selection was based on their relevance to the microstructural evolution and corrosion behaviour of the alloy. Figure 13a presents the TEM micrograph of the upper region, where no intermetallic phases are observed at the grain boundaries. In contrast, Figure 13c,d reveal both coherent and incoherent Nd-rich precipitates with acicular morphology at the grain boundaries. The classification of the precipitates as coherent or incoherent was based on their morphology and spatial relationship with the surrounding grains. According to Porter [47], precipitates located randomly within the matrix are typically incoherent, while those situated at grain boundaries and exhibiting partial alignment with one grain and mismatch with the other can be considered semi-coherent or coherent. Additionally, intermetallic precipitates rich in Al-Y-Nd-Gd with a plate-like morphology are also present at these boundaries. Figure 13f displays the TEM micrograph of the transverse section of the lower region, where spherical precipitates rich in Al-Y-Nd-Gd can be observed. This spherical geometry is produced as the precipitate tends to change its geometry towards a lower energy one [47]. During the manufacturing process, heat is conducted through the sample from the top towards the supports, resulting in prolonged exposure of the lower region to elevated temperatures compared to the rest of the sample. Consequently, in the middle region, Al-Y-Nd-Gd-rich precipitates exhibit a plate- or disk-like morphology, whereas in the lower region, they adopt a spherical shape. Extended thermal exposure facilitates solute migration to the grain boundaries, allowing the precipitates to grow towards a state of minimal interfacial free energy, thereby driving the morphological transition to a spherical form [47].

Figure 13b,e,g represents the EBSD maps, revealing grains with varying crystallographic orientations. Figure 13b, corresponding to the upper region, shows a grain size of 3.4 ± 1.4 µm with a slightly elongated morphology. In Figure 13e, representing the middle region, the grains exhibit an average size of 3.2 ± 1.3 µm and a nearly equiaxed morphology compared to the upper region. Additionally, grains with sizes close to 1 µm are observed, associated with a melt pool boundary. Figure 13g, depicting the lower region, displays a more homogeneous grain size distribution, with an average grain size of 3.8 ± 1.3 µm.

The differences in grain size and morphology do not appear to be a significant factor in the variation of the microhardness of the fabricated sample. However, the size and morphology of the grain boundary precipitates seem to have a more substantial influence. The successive laser passes during layer deposition likely promoted solute migration towards the grain boundaries, preventing the formation of other intermetallic compounds [34].

### 3.2. Electrochemical Test

#### 3.2.1. Linear Polarisation Resistance (LPR)

**Figure 14:**
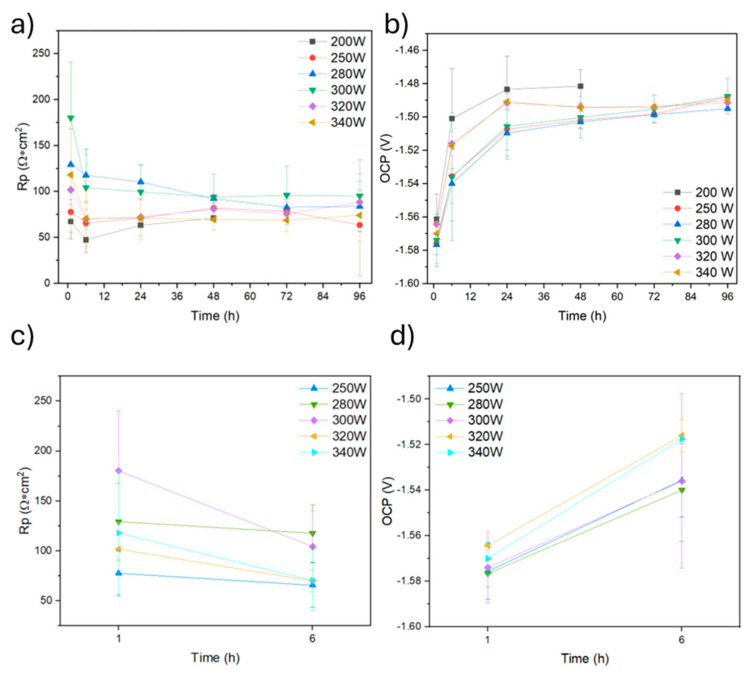
(**a**) Rp and (**b**) OCP of each specimen measured up to 96 h of immersion in 3.5% NaCl; (**c**) magnification of Rp up to 6 h; (**d**) magnification of OCP up to 6 h.

Figure 14a,c show the Rp results of the samples produced at 800 mm/s. At the beginning of the immersion time, a trend is observed indicating that samples obtained using intermediate and high powers exhibit the highest polarisation resistance. The highest average values were obtained for the 280 W and 300 W samples. As the immersion time progresses, polarisation resistance values for all samples tend to stabilise within the range of 50 to 100 Ω. This behaviour can be explained by the growth of a corrosion product layer on the surface of magnesium alloys, which is known to be only partially protective [12]. Thus, after 48 h, the 200 W sample was severely damaged, showing an accelerated corrosion process that can be explained by its high porosity, which could play a crucial role in decreasing corrosion resistance in two ways. Firstly, high porosity results in a significant increase in the specific surface area of the sample, and higher surface area in contact with the aggressive electrolyte can lead to accelerated corrosion. Furthermore, pores can act as a concentrator of chloride ions, potentially increasing the corrosion rate.

Figure 14b,d shows the evolution of the OCP potential of the specimens produced with a scanning speed of 800 mm/s. An initial increase of the corrosion potential values in all samples up to 24 h of immersion reflects the reactive character of magnesium. The evolution shows a stabilisation of the values over time, reaching a final value of approximately 1.49 V. When the laser power is increased, no significant variations of the corrosion potential are observed.

#### 3.2.2. Anodic–Cathodic Polarisation

**Figure 15:**
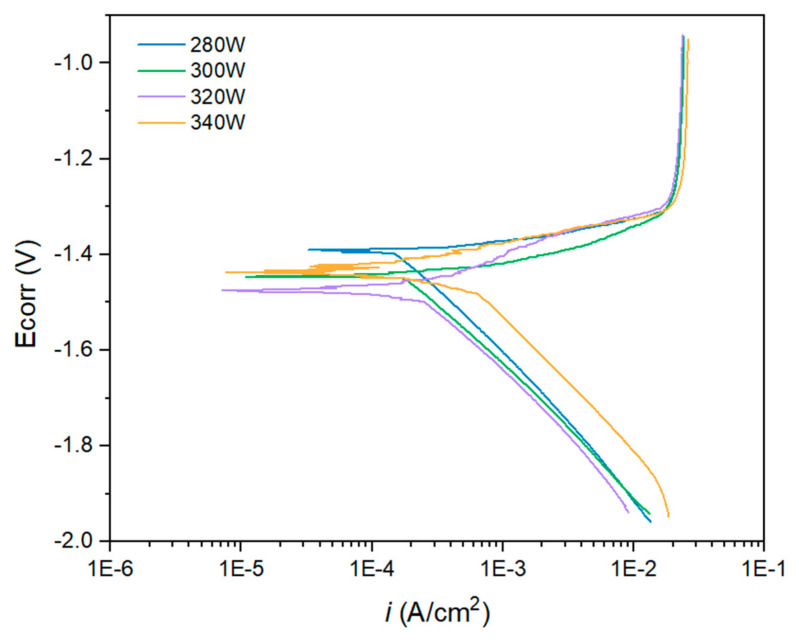
Anodic–Cathodic polarisation of each specimen was measured after 1 h of immersion in 3.5% NaCl.

Anodic–cathodic polarisation tests were exclusively conducted for the samples that exhibited the highest polarisation resistance values along the experimentation time, i.e., those with 280, 300, 320, and 340 W. Figure 15 and Table 3 present the results of the AC polarisation test. The Tafel extrapolation of the cathodic branch of the polarisation curves was employed to obtain the corrosion current density values for each sample [48].

Figure 15 shows the anodic–cathodic polarisation curves after 1 h of immersion in a 3.5 wt.% NaCl solution. The morphology of the A-C curves displayed in Figure 15 is typically obtained for magnesium alloys [12]. In this case, for all the samples, the E_corr_ potential value at which the anodic process increased exponentially, thus indicating an accelerated corrosion process, was very close to the E_OCP_ obtained during the stabilisation time previous to the A-C polarisation test. The samples obtained with a laser power of 280 and 300 W exhibited the best behaviour against corrosion as they presented the least electronegative open circuit potential values and the lowest values of current density. The current density increased as the power of the laser employed for the fabrication of the samples increased. Thus, the sample obtained with a laser power of 320 W showed a slightly higher corrosion current density compared with the previous samples, and the highest corrosion current density was obtained for the samples fabricated using a laser power of 340 W. However, the curves obtained for the higher laser power showed some structure in the vicinity of the corrosion potential, indicating that different phases could coexist.

The behaviour of the A-C curves displayed in Figure 15 could be explained by several phenomena. First, the low Tafel anodic slope for the WE43 magnesium indicates that this material is hardly polarisable as it is difficult to increase its potential without reaching excessive anodic current densities [12]. Moreover, magnesium alloys present some limitations during electrochemical tests due to the so-called negative difference effect (NDE) that takes place during corrosion of magnesium alloys [49,50]. This effect leads to imprecisions in the estimation of current density because the reaction mechanism around the corrosion potential induces changes in the polarisation curves, for example generating linear regions in the A-C curves. Finally, the instability of the corrosion products layer that precipitates on the surface of the metallic substrate, mainly composed of MgO and Mg(OH)_2_, prevents it to provide any effective protection against corrosion. However, for the WE43 alloy evaluated in this research, it has been suggested in the literature that Y and other alloying elements present in the matrix can incorporate to the corrosion products layer, increasing to some extent its stability and protective behaviour [33].

The corrosion current density values obtained from the A-C polarisation test for all conditions are higher than values found in the scientific literature for wrought and heat-treated AM WE43 samples [51], but in the same order of magnitude for additive manufactured samples [33]. WE43 samples obtained through traditional manufacturing processes exhibit microstructural differences compared with the samples obtained by additive manufacturing, and almost the absence of porosity, which can explain their improved corrosion resistance compared with samples obtained by additive manufacturing processes.

The samples evaluated in this research present different porosity, which is likely to play an important role in corrosion behaviour because of the surface area exposed, as well as differences at a microstructural level. Thus, as different factors affect the corrosion behaviour, *i*_corr_ was found slightly higher for the 300 W sample than for the 280 W one, despite of having lower porosity. However, this can be affected by the slightly higher corrosion potential. Also, in the case of the highest laser powers, the structure of the curves near the OCP can be attributed to the complex microstructure caused by the Marangoni forces that formed smaller structures that in the case of lower laser powers. This structure may locally condition the corrosion behaviour of the samples.

The severity of the corrosion process, in terms of depth of corrosion attack on the studied samples, was determined by considering the law of conservation of the electric charge, the electric charge (*Q*) transferred in the corrosion process is equal to the integral of the net current (*I*) generated by the anodic and cathodic reactions between time zero and the time elapsed in the process (Equation (2)). The oxidation reaction of magnesium in Equation (3) shows that for every mol of atoms of magnesium that are oxidised during the corrosion process, two mols of electrons are generated. Also, the first law of Faraday states that the charge *Q* transferred in the corrosion process can be calculated using Equation (4), where *F* is the Faraday constant and *N* are the mols of magnesium atoms oxidised during the corrosion process. Thus, substituting Equation (2) into Equation (4) leads to Equation (5).

(2) $$Q=\int_{0}^{t}I\;dt\;\;\;,$$

(3) $$Mg\to \;{Mg}^{2+}+2e^{-}\;,$$

(4) Q=2·N·F  ,

(5) $$N=\frac{\int_{0}^{t}I\;dt}{2\cdot F}\;.$$

The depth of the corrosion attack (*h*) can be correlated to the mass of the WE43 alloy that has undergone this attack. Equation (6) allows for the calculation of this mass based on the number of moles of Mg atoms oxidised during the process (*N*) and the molar mass of this element (*M_mol_*). This can be related to the depth of the attack through Equation (7). Substituting Equations (5) and (6) into Equation (7) leads to Equation (8), which allows for the calculation of the depth of corrosion attack as a function of the current density, which is a parameter that can be obtained from the anodic–cathodic polarisation tests.

(6) $$m=N\cdot M_{mol},$$

(7) $$m=\rho_{Mg}\cdot A\cdot h\;,$$

(8) $$h=\frac{M_{mol}}{2\;\rho_{Mg}\;F}\int_{0}^{t}i_{corr}\;dt.$$

Moreover, the current density of the system is related with the polarisation resistance (*R_p_*) through the Stern–Geary equation (Equation (9)), which correlates these two magnitudes by the parameter *B*, that is calculated from the slopes of the anodic and cathodic branches from the Evan’s diagram obtained during the anodic–cathodic polarisation tests as shown in Equation (10). While it is true that the negative difference effect of magnesium alloys could negatively affect the precision of the measurements, the deviation would be similar for all the samples. Thus, assuming that the Tafel slopes are constant for the different immersion times, the depth of corrosion was calculated using Equation (11) and the values are shown in Table 4. In the end, the values calculated for the depth of the corrosion attack were significantly lower for the samples that also presented the lowest current density values, those obtained with a laser power of 280 and 300 W. Based on the calculated values, these samples would present a corrosion rate of 0.17 mm/y and 0.27 mm/y, respectively.

(9) $$i_{corr}=\frac{B}{R_{p}},$$

(10) $$B=\frac{\beta_{anodic}\cdot \beta_{cathodic}}{2.3\cdot \left(\beta_{anodic}+\beta_{cathodic}\right)},$$

(11) $$h=\frac{M_{mol}\;B}{2\;\rho_{Mg}\;F}\int_{0}^{t}\frac{dt}{R_{p}}\;\;\;$$

#### 3.2.3. Electrochemical Impedance Spectroscopy (EIS)

**Figure 16:**
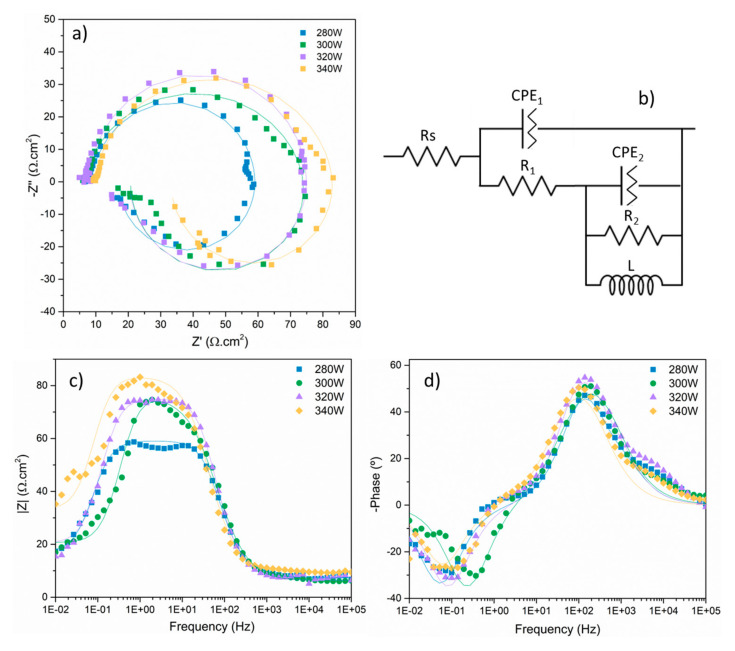
Nyquist (**a**) and Bode plots (**c**,**d**), and equivalent circuit (**b**) of each specimen measured after 1 h of immersion in 3.5% NaCl.

Figure 16 shows the Nyquist and Bode plots obtained after electrochemical impedance spectroscopy tests for each sample condition after 1 h of immersion in 3.5% NaCl electrolyte. For all conditions, Nyquist curves present the typical morphology for magnesium alloys [12], exhibiting two time constants represented by two capacitive loops at high and medium frequencies, and an inductive loop at low frequencies. For all cases, the curves can be modeled using the equivalent circuit displayed in Figure 16b. In this circuit, *R*s represents the electrical resistance of the electrolyte. The corroding samples did not show perfect capacitive behaviour, as can be seen in Figure 16d, where phase angles were always greater than −90°. Thus, constant phase elements instead of capacitors were used for the numerical fittings [52]. Table 5 shows the values of the different circuit elements that best fit the EIS curves. *CPE*_1_ and *R*_1_ form the first-time constant associated with the capacitive loop at high frequencies. Generally, this time constant is associated with the formation of a corrosion products layer that precipitates on the surface of the metallic substrate and, in the case of magnesium, offers only partial protection against corrosion, especially considering the generation of H_2_ bubbles, inherent to the corrosion process of magnesium, that increases the porosity of the corrosion products layer, facilitating the intake of electrolyte and making it less protective. Thus, the amplitude of the capacitive loop is much lower compared with magnesium alloy samples treated with a coating treatment that offers high corrosion protection to the metallic substrate [53]. *CPE*_1_ and *R*_1_ can be, respectively, correlated with the capacitance of the corrosion products layer formed on the surface of the metallic substrate and the resistance offered by the porous corrosion products layer and the subsequent electrolyte intake. The second time constant represented in Figure 16b by the constant phase element *CPE*_2_ and the resistor *R*_2_ represents the capacitive loop at medium frequencies and can be associated with charge transfer and degradation phenomena taking place at the corrosion products layer–substrate interface once the electrolyte passed through the corrosion products layer and reached the metallic substrate, promoting the formation of new corrosion products and also involving the mass transfer of Mg^2+^ ions through the corrosion products layer [52].

The presence of the inductive loop at low frequencies, represented in the electrical circuit in Figure 16b by inductor *L*, has been described in scientific literature, with some authors relating it to an accelerated anodic dissolution of magnesium or the progression of pitting corrosion on the surface of magnesium substrates [54]. Also, the inductive loop has been associated with adsorption/desorption processes due to the relaxation of metallic ions on the surface of the substrate, related to charge transfer phenomena. At low frequencies, these ions have enough time to interact with the surface of the substrate [55]. Moreover, the presence of an inductive loop can be indicative of morphological changes in the surface of the corroding substrate [55].

The data obtained from the EIS tests indicate that the behaviour against corrosion of the different conditions is similar, with very small differences between samples in terms of impedance. At high frequencies, all the samples present the same behaviour, indicating the same limited protection provided by the corrosion products layer that precipitates on the surface of the samples.

For short immersion times (1 h), 300 W samples present the highest polarisation resistance values, while 280 W samples show similar values and even better after 6 h of immersion. Also, these samples present one of the lowest current density values from the anodic–cathodic polarisation tests. This behaviour could be related to the fact that these samples present one of the lowest porosity of all samples fabricated in this research, as porosity can negatively affect the corrosion behaviour of the samples as pores increase the specific area of the sample exposed to the aggressive electrolyte, increasing the degradation rate, especially in the case of materials whose corrosion products do not form a protective layer, as in the case of magnesium alloys. However, porosity does not appear to be the determining factor in this case since significant differences were found in terms of porosity between the studied samples, but all samples exhibit corrosion resistance values in the same order of magnitude and closely aligned, as indicated by the electrochemical test results. Additionally, the high laser power used in sample fabrication does not introduce comparative issues, since no significant differences in corrosion resistance were observed between samples produced with varying laser powers (280, 300, 320, and 340 W). Finally, considering the aggressiveness of the electrolyte used in the corrosion tests, the results obtained in this research are consistent with the information found in the literature regarding the corrosion behaviour of WE43 samples obtained by additive manufacturing [32,33], considering the behaviour of samples of the same alloy tested in less aggressive environments.

In the overall corrosion behaviour assessment, considering not only the EIS data but also the polarisation resistance and the anodic–cathodic polarisation tests, the results indicate that the samples obtained using a laser power of 300 W and a laser scanning speed of 800 mm/s present the best performance when looking for balanced corrosion behaviour both for short and long immersion times, presenting a reduction in current density from the AC test of about 56% compared to the sample with the highest current density value, and an increase in the resistance to charge transfer, obtained from the EIS tests, of about 25% compared to the sample with the lowest resistance against corrosion.

The DC testing behaviour indicates that samples produced at lower laser powers, such as 280 W, exhibit superior performance compared to those fabricated at higher power levels. However, this difference becomes less significant when the porosity content is below 1%, suggesting that porosity may not have a significant long-term impact.

In the case of AC testing, the R_1_ values obtained from EIS show improved performance when using powers greater than 280 W. This may be attributed to the formation of a more resistive oxide layer at higher power levels, which initially blocks the pores. Additionally, the values for charge transfer resistance (R_2_) reach a maximum at 300 W, then decrease as the power increases further. This variation in AC test behaviour could be related to the microstructural changes observed in the samples. At higher power levels, the molten pool becomes increasingly unstable, promoting the formation of Marangoni convection currents. These currents modify the cooling dynamics, resulting in the dilution of precipitates within the alloy and the development of oxide films around pores and lack-of-fusion defects. This morphology causes a more complex corrosion behaviour, which, along with the closeness of the OCP to the pitting potential, explains the differences observed between the different conditions.

## 4. Conclusions

In this study, dense samples of WE43 magnesium alloy were produced using Laser Powder Bed Fusion (LPBF). The process parameters were evaluated to determine their influence on sample density. Additionally, a microstructural analysis was performed, followed by an electrochemical analysis in a 3.5 wt.% NaCl solution.

- The lowest porosity (0.1%) was obtained for 300 W laser power and 800 m s^−1^. Higher laser power caused material evaporation, and higher scanning speeds caused porosity.
- Microhardness values were ~20% higher than for the same alloy produced by casting and were not significantly dependent on the manufacturing conditions. Microhardness was higher at the top of the sample because consecutive heat cycles favored incoherent precipitate deposition.
- Nd precipitates exhibit coherent and incoherent interfaces and needle-like morphology at grain boundaries. Al-Y-Nd-Gd-rich precipitates have a plate-like morphology in the middle zone and are spherical in the lower zone.
- The samples with the lowest porosity, and particularly the 300 W 800 m s^−1^ one, exhibited the best corrosion behaviour for short and long immersion times. Electrochemical impedance spectroscopy (EIS) showed that high laser power samples showed different corrosion mechanisms than the optimal ones because the different melting processes change the structure of the oxides formed.
- In summary, the optimal AM WE43 magnesium alloy samples were dense and had higher hardness and slightly lower corrosion resistance than conventionally manufactured and heat-treated WE43 samples.

## Figures and Tables

**Table 1:** Composition in wt.% of WE43 magnesium alloy powder.

Mg	Y (%)	Nd (%)	Gd (%)	Zr (%)	Zn (%)	Al (%)	Fe (%)
Balance	3.76	2.46	1.26	0.40	0.21	<0.05	0.0072

**Table 2:** Manufacture parameters of LPBF process.

Power (W)	200–250–280–300–320–340–360–380–400
Scan speed (mm/s)	800–1000–1200

**Table 3:** E_corr_ and corrosion current density values extracted from A-C polarisation curves in Figure 15.

Laser Power	E_corr_ (V)	*i_corr_* (A/cm^2^)
280 W	−1.38	1.7 × 10^−4^
300 W	−1.43	1.9 × 10^−4^
320 W	−1.47	2.2 × 10^−4^
340 W	−1.41	4.3 × 10^−4^

**Table 4:** Calculated depth of corrosion after different immersion times.

Corrosion Depth (μm)					
Laser Power	1 h	6 h	24 h	48 h	72 h
280 W	0.01	0.07	0.28	0.69	1.39
300 W	0.01	0.12	0.60	1.36	2.38
320 W	0.11	1.24	4.31	8.07	12.1
340 W	0.22	2.03	7.36	13.0	19.9

**Table 5:** Values for EIS circuit fitting.

Laser Power	Rs (Ω·cm^2^)	CPE_1_ (S·s^n^/cm^2^)	n_1_	R_1_ (Ω·cm^2^)	CPE_2_ (S·s^n^/cm^2^)	n_2_	R_2_ (Ω·cm^2^)	L (H·cm^2^)
280 W	26.1	8.6 × 10^−6^	0.99	34.8	10.3 × 10^−6^	0.99	160	239
300 W	22.9	21.8 × 10^−6^	0.90	51	51.8 × 10^−6^	0.55	200	70
320 W	23.1	9.9 × 10^−6^	0.99	50	7.4 × 10^−6^	1.00	194	197
340 W	33.5	33.8 × 10^−6^	0.90	85	1 × 10^−6^	0.80	180	290

## Data Availability

The original contributions presented in this study are included in the article. Further inquiries can be directed to the corresponding author.
